# Self-organized ventilation defects in asthma: insights and open-source implementation of the Venegas–Winkler model

**DOI:** 10.1098/rsos.261168

**Published:** 2026-10-07

**Authors:** Graham M. Donovan

**Affiliations:** ^1^ Department of Mathematics, The University of Auckland, Auckland, New Zealand

**Keywords:** biomechanics, mathematical modelling, computational biology, bronchoconstriction, ventilation heterogeneity, airway tree, bistability, reproducibility

## Abstract

The Venegas–Winkler model is a seminal model in asthma pathophysiology, providing both a conceptual and quantitative framework for extending a posited bistability between open and closed states at the level of the individual, terminal airway to the lung as a whole. In doing so, it offers an explanation for the characteristic ventilation heterogeneity (or clustered ventilation defects) seen in asthma. The terminal airway bistability is based on the Anafi–Wilson model, and this is embedded and extended into a symmetric bifurcating airway tree, in which an enforced tidal volume target and an imposed degree of airway smooth-muscle activation interact to generate spatially structured ventilation patterns. Here, we provide the first open-source implementation of the Venegas–Winkler bronchoconstriction cascade model and use it to identify several previously undescribed properties: both the tree depth and wall thickness assumptions affect the bifurcation onset, tidal volume and acinar elastance assumptions require careful interpretation for a model at sub-lung scale and airway geometry assumptions strongly affect driving pressures and bifurcation cascade characteristics.

## 1. Introduction

The Venegas–Winkler model [1] is a seminal model in asthma pathophysiology, making concrete and accessible the consequences of bistability at the airway level and the ways that this can manifest as self-organized ventilation patterns at the lung scale, with important consequences for understanding both the underlying physiology and also treatments. The original motivation is straightforward: begin with the terminal airway model (and attendant bistability) of the Anafi–Wilson model [2] and extend this into a (symmetric) airway tree. The original paper, as dictated by the format (letter to *Nature*), was relatively sparse on model details, but provided an overview in the supplementary material. However, despite being used in at least a dozen subsequent studies [3–14], full model details, including parameter values and numerical methods, have never been published, and the model has never been reproduced outside of the original research group and codebase.

Here, we provide the first open-source implementation of the Venegas–Winkler bronchoconstriction cascade model (VW05) and use it to identify several previously undescribed properties: both the tree depth and wall thickness assumptions affect the bifurcation onset, tidal volume and acinar elastance assumptions require careful interpretation for a model at sub-lung scale and airway geometry assumptions strongly affect driving pressures and bifurcation cascade characteristics.

Although a separate lineage of models based on similar ideas [15–18] is also available in the literature, the publication of a publicly available, open-source interpretation of the VW05 model may serve to increase its impact. The original paper is widely cited outside of physiology, for example in the tipping points and critical transitions literature [19–23]; the model's impact would likely be greater if it could be practically used by others.

## 2. Methods

### 2.1. Model statement

#### 2.1.1. Anafi–Wilson terminal airway bistability

The single-airway dynamics on each branch follows the Anafi–Wilson model [2]. The original paper provides a complete description of the (single) terminal airway model, and this has previously been reproduced [24,25]. However, in the interests of completeness, the governing equations are restated here.

Let $\rho_{m}$ denote the dimensionless smooth-muscle radius and $\rho_{i}$ the dimensionless inner (luminal) radius of an airway, normalized by the relaxed outer radius $r_{o}$. The wall is treated as incompressible, with a wall area cross-sectional fraction f, giving the geometric constraint

(2.1) $$\rho_{i}^{2}=\rho_{m}^{2}-f.$$

The static force balance on the airway wall in [2] reads

(2.2) $$\frac{T}{r_{o}\rho_{m}}=P_{\text{tm}},$$

where T is the hoop stress in the airway wall and $P_{\text{tm}}$ is the transmural pressure. The active smooth-muscle tension follows a Gunst–Stropp-style linear length–tension curve,

(2.3) $$\frac{T_{\max}}{T_{o}}=\tau_{r}\;(1.25\rho_{m}-0.25),$$

where $T_{\max}$ is the maximum of T over the breathing cycle, $T_{o}/r_{o}=40\;{\text{cmH}}_{2}\text{O}$ and $\tau_{r}\in [0,1]$ is a global control parameter for airway smooth-muscle activation. Here, the constants were estimated by Anafi & Wilson [2] as an approximation of the isometric force–length curve [2, fig. 2]. Combining equations (2.2) and 2.3 gives the muscle line we invert at the end of each breath:

(2.4) $$P_{\text{tm}}^{\max}=\frac{T_{o}\tau_{r}}{r_{o}}\left(1.25-\frac{0.25}{\rho_{m}}\right),$$

where $P_{\text{tm}}^{\max}$ denotes the per-breath peak of $P_{\text{tm}}$ tracked during the within-breath integration.

The peribronchial pressure $P_{\text{pb}}$ is the alveolar pressure plus the [26] tethering correction:^1^

(2.5) $$P_{\text{pb}}=P_{A}\left(1+1.4x+2.1x^{2}\right),\;\text{where}\;\;\;x=1-\frac{\rho_{m}}{v^{1/3}},$$

v is the local parenchymal volume normalized to total lung capacity and $P_{A}$ is the alveolar pressure. Here, the constants are owing to Lai-Fook [26], based on theoretical large-deformation solutions for symmetric inflation of a uniform hole in parenchyma and calibrated to data from isolated dog lobes [26, fig. 8]. The transmural pressure is then

(2.6) $$P_{\text{tm}}=P_{\text{lumen}}-P_{A}+P_{\text{pb}}=P_{\text{lumen}}+P_{A}g(x),\;\text{where}\;\;\;g(x)=\left\{ \begin{array}{ll} 1.4x+2.1x^{2}, & x>0,\\ 0, & x\leq 0 \end{array}\right.$$

and $P_{\text{lumen}}$ is the lumen pressure.

Airway resistance $R_{\text{aw}}$ follows a modified version of Poiseuille flow:

(2.7) $$R_{\text{aw}}=\frac{12\mu \ell }{\pi (r_{o}\rho_{i}{)}^{4}},$$

with $\mu =1.81\times 10^{-4}$ poise (air viscosity at body temperature) and ℓ the airway length. Here, the factor of 12 in place of the standard 8 is intended to capture small additional flow-area losses from the wall folding [27]; certainly other approaches are possible and justifiable [28,29] but here we confine ourselves to the stipulations of the original Anafi–Wilson model as inherited by VW05.

The airway network is a symmetric dichotomously branching tree of $N_{g}$ generations of bifurcation,^2^ so it contains $2^{N_{g}+1}-1$ airways and $2^{N_{g}}$ terminal acini. Venegas *et al.* [1] stipulate ‘a symmetric tree with 12 generations of bifurcation (anatomic generations 4 to 16 in the Weibel model)’. This sets $N_{g}=12$, $N_{\text{seg}}=8191$, $N_{\text{term}}=4096$ and identifies the anatomic Weibel generations from which the airway lengths and diameters are taken.

### 2.2. Embedding the Anafi–Wilson model into an airway tree

Now, we must embed the Anafi–Wilson model into the tree topology. The following aspects are specified in [1] or follow-on papers:

— Each terminal airway subtends an acinar unit with linear parenchymal compliance (acinar elastance $E_{\text{unit}}$).
— The system is ventilated with positive end-expiratory pressure (PEEP), constant inspiration and passive exhalation. Wongviriyawong *et al.* [4] and Wongviriyawong [7] use volume-controlled ventilation (VCV) with constant inspiratory flow followed by passive expiration to PEEP. Tidal volume is 600 ml, frequency is 12 breaths min^–1^, I : E ratio is 1:1, PEEP is 5 cmH${}_{2}$O.
— Small symmetry-breaking perturbations are added to the wall thickness (approx. 1% coefficient of variation).
— Smooth-muscle activation $\tau_{r}$ varies between 0 and 1.
— The system is integrated over many breaths with an explicit forward Euler integrator with 10 ms time step [11]. The airway radii are adjusted breath-to-breath towards the peak-transmural-pressure value implied by the muscle force balance.
— Asymmetric speed constants are used for dilation and contraction [9].

*Within-breath ventilation and integrator.* We adopt VCV with these settings; we use Δt=10 ms throughout. However, with the Anafi–Wilson per-acinus parameters discussed below, the terminal-acinus time constant $\tau =R_{\text{term}}/E_{\text{unit}}$ falls below Δt at moderately dilated states, making explicit forward Euler borderline stable and, in our experiments, prone to runaway. We instead use a single *backward (implicit) Euler* step at the terminals at each Δt. Concretely, at each time step, we augment the terminal resistance by $\Delta t\cdot E_{\text{unit}}$,

(2.8) $$R_{\text{term}}^{\text{impl}}=R_{\text{term}}+\Delta t\cdot E_{\text{unit}},$$

and use this in the network solve; the Thévenin source^3^ at each terminal is the current alveolar pressure. The volume update is then

(2.9) $$V_{\text{unit}}^{n+1}=V_{\text{unit}}^{n}+\Delta t\cdot Q_{\text{term}},$$

where $Q_{\text{term}}$ is the flow out of the network solve. This preserves mass conservation exactly: the trunk flow boundary condition implies $Q_{\text{trunk}}=\sum_{t}Q_{\text{term},t}$ at every step. It is unconditionally stable, at the cost of a slight additional dissipation of order Δt.

The tree itself is solved as a linear network at each step. With all branch resistances frozen for the duration of the breath, the algorithm is:

— Compute each terminal airway's alveolar pressure $P_{A}=E_{\text{unit}}(V_{\text{unit}}-V_{\text{RV}})$.
— Walk the tree leaves-to-root, replacing each subtree by a Thévenin pair $\left(R_{\text{eff}},P_{\text{eff}}\right)$: for two daughters with $\left(R_{a},P_{a}\right)$ and $\left(R_{b},P_{b}\right)$ joined to a parent of resistance R, the parent subtree's pair is $R_{\text{eff}}=R+R_{a}R_{b}/(R_{a}+R_{b})$ and $P_{\text{eff}}=(P_{a}R_{b}+P_{b}R_{a})/(R_{a}+R_{b})$.
— Apply the trunk boundary condition (constant $Q_{\text{trunk}}$ during inspiration; mouth pressure equal to PEEP during expiration) to obtain trunk flow and inlet pressure.
— Walk root-to-leaves to distribute flow into each subtree using the stored Thévenins.

This is $O(N_{\text{seg}})$ per step and uses no global matrix solve. The sequential P–Q update structure matches the description in [4]: ‘…radii fixed within a breath, pressure and flow solutions iterated…’.

*Breath-to-breath update.* Golnabi *et al.* [9] state the update rule explicitly. For each airway,

(2.10) $$\rho_{m}^{\left(b+1\right)}=\rho_{m}^{\left(b\right)}+S_{x}\;\left(\rho_{m}^{*,(b)}-\rho_{m}^{\left(b\right)}\right),\;S_{x}=\left\{ \begin{array}{ll} S_{c}, & \rho_{m}^{*}<\rho_{m},\\ S_{d}, & \rho_{m}^{*}>\rho_{m}, \end{array}\right.$$

where $\rho_{m}^{*}$ is the target radius implied by the current breath's peak transmural pressure via equation (2.4), and the asymmetric speed indices are $S_{c}=0.05$ (constriction) and $S_{d}=0.33$ (dilation). These values are taken directly from [9] and were in turn based on estimates of smooth-muscle kinetics in [31,32].

#### 2.2.1. Implementation of the Anafi–Wilson model at scale

There are now choices to be made about the numerical solution procedure for the Anafi–Wilson model at scale. In [2], the procedure described (explicit curve intersection finding) is sufficient for demonstration purposes with a single airway, but not suitable for extension to the tree geometry from either a computational cost or numerical robustness point of view.

We are then left to infer the likely numerical method. There appear to be two likely options: fixed-point iteration, or explicit solution of the underlying cubic with branch assignment. We tested both approaches and found similar results, but that the former is more likely to be consistent with the computational cost of the original implementation. Thus, inverting equation (2.4) for $\rho_{m}$ gives

(2.11) $$\rho_{m}^{*}=\frac{0.25\;T_{o}\tau_{r}/r_{o}}{1.25\;T_{o}\tau_{r}/r_{o}-P_{\text{tm}}^{\max}},$$

where the denominator is non-positive (very high $P_{\text{tm}}^{\max}$), the muscle cannot hold against the distending force and we set $\rho_{m}^{*}=1$. A numerical floor is also imposed as a regularizer to prevent resistance runaway at closure.

*Peak transmural pressure during a breath.* Anafi & Wilson [2] compute $P_{\text{tm}}^{\max}$ as the maximum over the breath cycle, with $P_{A}$ and v covarying with the within-breath dynamics, and we do the same: at every time step, we evaluate 2.6 per branch and track the per-branch maximum across all steps in the breath. The peak typically occurs at end-inspiration under VCV.

*Internal-airway quantities.* For *internal* (non-terminal) airways, Venegas *et al.* [1] and the follow-ups do not state how $P_{A}$ and v in equation (2.5) are computed. The natural choice, and the one used here, is to average over the subtree that the airway subtends: a non-terminal airway's external alveolar pressure is taken to be the mean alveolar pressure of all terminal units it serves, and its local v is computed from the mean acinar volume of those same terminals. The mean is computed bottom-up by tree traversal:

(2.12)Vsubtree-avg(seg)=1Nsubtended∑t∈subtreeVunit,t(2.13)andPAlocal=Eunit(Vsubtree-avg−VRV).

*Symmetry breaking.* Venegas *et al.* [1] state that the symmetry of the tree is broken by a small (approx. 1% CV) Gaussian perturbation in wall thickness, frozen for the duration of a simulation. We implement this by perturbing f directly with 1% CV; the effect on the relaxed lumen and resistance is to leading order linear in the perturbation, and matches the published prescription.

*Chest-wall compliance.* Wongviriyawong *et al.* [4] drop chest-wall compliance ($C_{\text{cw}}\to \infty$). Venegas *et al.* [1] retain it in their supplement but do not specify a value. We follow [4] and neglect it.

*Initial state and the $\tau_{r}=0$ ramp.* We start with all airways at $\rho_{m}^{\left(0\right)}=1$ and all acini at $V_{\text{unit}}=V_{\text{RV}}+\text{PEEP}/E_{\text{unit}}$, so that the first inspiration begins from mouth pressure equal to PEEP and zero flow. The protocol then runs an initial fast ramp of $\tau_{r}$ from 0 to 0.5 over 50 breaths, followed by a long hold at $\tau_{r}=0.5$, ramp up to $\tau_{r}^{\text{peak}}$ and hold there.

The $\tau_{r}=0$ phase is not a true Anafi–Wilson equilibrium: with $\tau_{r}=0$, the muscle line is identically zero and the equilibrium condition $\text{muscle}(\rho_{m})=P_{\text{tm}}^{\max}(\rho_{m})$ has no solution on $\left[\rho_{m}^{\min},1\right]$ because the right-hand side is strictly positive. The simplified [1]/[2] formulation carries only an active muscle force and no passive-wall stiffness, so at $\tau_{r}=0$, there is no restoring force to limit dilation and $\rho_{m}$ would run to infinity. We interpret the missing physics as a passive-wall stiffness that fixes the airway at its natural radius when the muscle is inactive, and implement it as a hard clamp: the target $\rho_{m}^{*}$ in the breath-to-breath update is clamped to $\rho_{m}^{*}\leq 1$. With this clamp, $\rho_{m}$ sits at 1 throughout the $\tau_{r}\approx 0$ phase, defining the baseline $r_{0}$ that Wongviriyawong *et al.* [4] use to normalize all subsequent radii.

The plateau at $\tau_{r}=0.5$ is then a true Anafi–Wilson equilibrium and settles to $\rho_{m}\approx 0.85$ within a few tens of breaths from the $\tau_{r}=0$ baseline.

*Reproduction choices and imputed parameter values.* After consulting [1–4,6,7,9,11,13,25,26], the following choices are imputations and not directly supported by the literature:

— Internal-airway tethering averaging. The choice of mean subtended-acinar volume for $P_{A}^{\text{local}}$ and v at internal airways is the natural reading of Venegas *et al.* [1] but is not explicitly stated in any of the papers consulted.
— Passive-wall clamp on $\rho_{m}$. The Anafi–Wilson formulation has no explicit passive-wall stiffness; at $\tau_{r}=0$, the muscle force is identically zero and the airway has no restoring force against dilation. We clamp the breath-to-breath target $\rho_{m}^{*}\leq 1$ to represent the passive-wall fixing the airway at its natural radius. We also impose a numerical floor $\rho_{i}\geq 0.005$, which is simply a numerical regularizer to keep R finite. The qualitative dynamics are not sensitive in this range.
— Whether and how chest-wall compliance is included.
— Selection of the specific Weibel table values.

Table 1 lists all the constants used. Where a value comes directly from a cited source, we mark it; where it is imputed, we say so. The parameter regime is also constrained by the assumption of marginal explicit forward Euler stability at 10 ms time step.

**Table 1.** Parameter values and other methodological assumptions used in this reproduction.

	value	description	source
$N_{g}$	12	generations in airway tree	[1]
$N_{\text{seg}}$	8191	total airways	[1]
$N_{\text{term}}$	4096	terminal acini	[1]
$T_{o}/r_{o}$	40 cmH${}_{2}$O	isometric muscle stress per relaxed radius	[2]
f	0.26 (1% CV)	wall area cross-sectional fraction	imputed^5^
$E_{\text{unit}}$	77.5 cmH${}_{2}$O ml^–1^	per-acinus elastance	imputed^6^
$V_{\text{TLC}}^{\text{unit}}$	$25/E_{\text{unit}}\approx 0.30$ ml	per-acinus total lung capacity	[2] calibration
$V_{\text{RV}}^{\text{unit}}$	$0.2\;V_{\text{TLC}}^{\text{unit}}$	per-acinus residual volume	[2]
$V_{T}^{\text{unit}}$	0.147 ml acinus^–1^	per-acinus tidal volume (total 600 ml)	[4,7]
μ	$1.81\times 10^{-4}$ poise	gas viscosity (air, body temperature)	standard
RR	12 min^–1^	breathing rate	[4,7]
I : E	1 : 1	inspiration : expiration ratio	[4,7]
PEEP	5 cmH${}_{2}$O	positive end-expiratory pressure	[4,7]
integrator	implicit Euler^7^	within-breath update	imputed
$S_{c},S_{d}$	0.05, 0.33	constriction and dilation speeds (per breath)	[9]
$\rho_{i}^{\min}$	0.005	numerical floor on inner radius	assumed
$\rho_{m}^{\left(0\right)}$	0.85	initial muscle radius (relaxed)	imputed
$\rho_{m}^{\max}$ upper limit	1.0	passive-wall ceiling on target $\rho_{m}^{*}$	imputed

There are several places where the imputed parameters differ from what might be naively assumed, and these are worth emphasizing as they organize the model behaviour in important ways. One is the wall area cross-sectional fraction f: inheriting the stipulated value from Anafi–Wilson suggests f=0.16, whereas we find that the location of the (mathematical^4^) bifurcation onset is near $\tau_{r}\approx 1.1$, rather than the ≈0.85 in the original papers and reproduced here. Thus, the assumed value of airway wall thickness is an important consideration in organizing the model behaviour, especially in terms of bifurcation onset. Another is that the airway geometry must be scaled up from the ‘standard’ Weibel values, which are estimated at approximately 75% TLC, in order to give estimates of the TLC values for use in the model. (Otherwise the driving pressures are far too large, and no closure cascade is possible.) Because these scaled values are not explicitly stated anywhere in the literature, we have taken the simplest possible approach and scaled them linearly. A nonlinear approach would almost certainly be more accurate and justifiable, but in the absence of specific information about how this was done originally, the search space is simply too large. Finally, the per-unit elastance $E_{\text{unit}}$ must be reconciled with the scale of the lung model; we will revisit this issue in the Discussion.

*Airway geometry.* We adopt the standard [33] values for the anatomic generations 4–16 (table 2). Airway i has daughters at indices 2i and 2i+1 in one-based indexing (or 2i+1, 2i+2 in zero-based indexing); the trunk is index 1, and the terminals occupy indices $2^{N_{g}}\ldots 2^{N_{g}+1}-1$.

**Table 2.** Weibel (1963) model A mean airway diameters for generations 4–16, as measured at approximately 75% TLC. In the Anafi–Wilson (2001) and the Venegas–Winkler lineage, these are treated as the fully distended (TLC) calibre. The $d_{\text{eff}}$ column gives the scaled values used in this work (1.35×d), chosen to match the published mean $P_{ao,p}$ at the operating $E_{\text{unit}}$. Length ℓ is from Weibel and is used as published.

generation	d (original Weibel, approx. 75% TLC, mm)	$d_{\text{eff}}$ (scaled, mm)	ℓ (mm)
4	4.50	6.075	12.7
5	3.50	4.725	10.7
6	2.80	3.78	9.0
7	2.30	3.105	7.6
8	1.86	2.511	6.4
9	1.54	2.079	5.4
10	1.30	1.755	4.6
11	1.09	1.4715	3.9
12	0.95	1.2825	3.3
13	0.82	1.107	2.7
14	0.74	0.999	2.3
15	0.66	0.891	2.0
16	0.60	0.81	1.65

*Implementation.* The model is implemented in MATLAB (with Octave compatibility) and is available at http://doi.org/10.5281/zenodo.20387023. A Python port is also available. Both implementations share the same algorithm, give the same numerical results to several decimal places where the random-number generator (RNG) is matched and produce the same qualitative bifurcation behaviour without RNG matching, with comparable runtimes. The main computational cost is per-step network solve, and on *ca* 2025 desktop hardware the simulation wall time is approximately 3 minutes.^8^

## 3. Results

We selected two reproduction targets from the lineage of VW05 papers: (i) the detailed VCV time series and ventilation plots in [4, fig. 2], reproduced here in figure 1, and (ii) the generation and bifurcation dependent detail in [3, fig. 1], reproduced here in figure 2. From these, there are several important qualitative and quantitative features to be reproduced:

— There is an $r/r_{0}\equiv 1$ (homogeneous) state at $\tau_{r}=0$.

**Figure 1.**
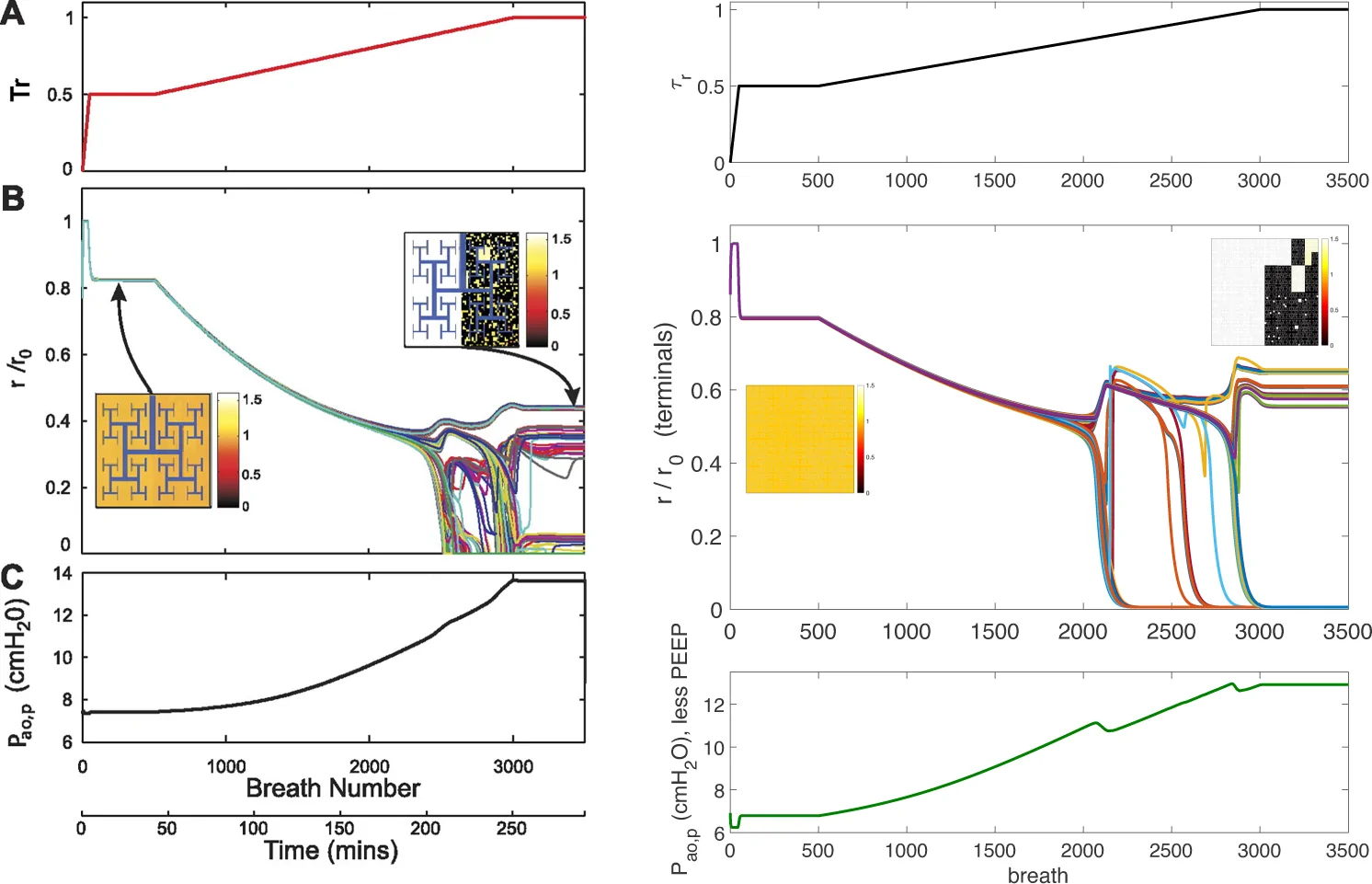
Comparison between (left) reproduction target ([4, fig. 2], reproduced with permission) and (right) model code output (reproduction simulation, created with VW05(12,1) from code at http://doi.org/10.5281/zenodo.20387023). (A) The imposed ramp in smooth-muscle activation; note that Tr in the original notation is equivalent to our $\tau_{r}$. (B) The resulting airway radius time course for the terminal airways only, in response to the imposed stimulus; in particular, observe that the radii constrict homogeneously up to the bifurcation onset at $\tau_{r}\approx 0.85$. The insets show the spatial distribution of flow during the initial and final plateaus. (C) The airway entrance pressure (less PEEP) required to maintain the target tidal volume in response to the imposed airway smooth-muscle constriction.

**Figure 2.**
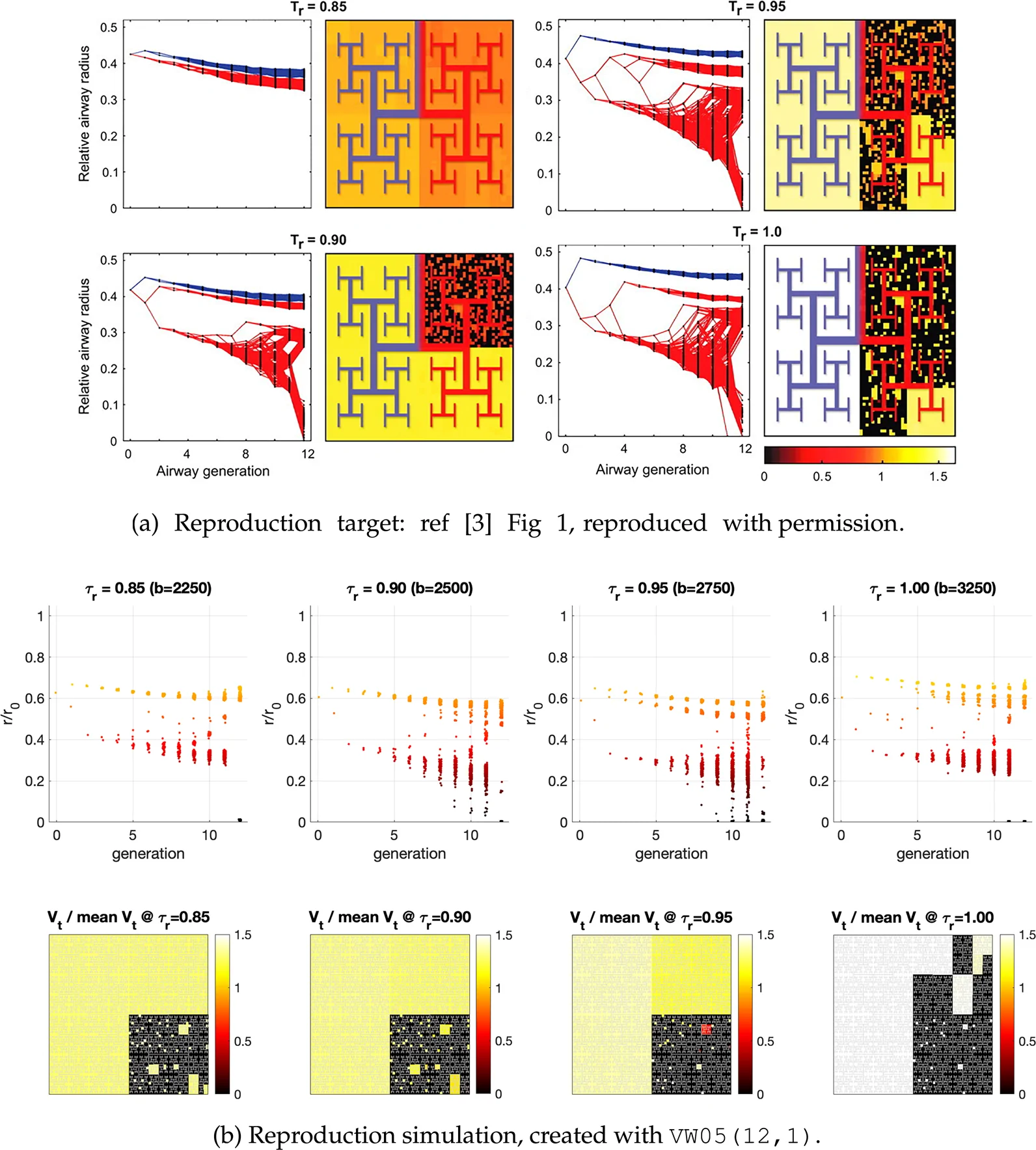
Constriction pattern by generation, and dependence on smooth-muscle activation ($\tau_{r}$). Comparison between (a) reproduction target ([3, fig. 1], reproduced with permission) and (b) model code output (reproduction simulation, created with VW05(12,1)). In each pair of panels, one panel shows the relative airway radius ($r/r_{0}$) versus airway generation, and the other panel shows the spatial ventilation distribution, as the smooth-muscle activation level is varied. Note that Tr in the original notation is equivalent to our $\tau_{r}$. Observe that full closure occurs predominantly at the terminal generation, and that the intermediate-value branch structure is associated with clusters of different sizes.

— There is a plateau at $r/r_{0}\approx 0.82$ (homogeneous) at $\tau_{r}=0.5$.
— Homogeneous descent occurs through the $\tau_{r}$ ramp, to a bifurcation occurring at $\tau_{r}\approx 0.85$ and $r/r_{0}\approx 0.4$.
— There is a bifurcation cascade resulting in clustered ventilation defects, in particular a uniformly open-branch half, and a dominantly closed half with scattered open regions of different sizes within.
— Top-of-tree pressure rises from about 7 ${\text{cmH}}_{2}\text{O}$ at baseline to about 13 ${\text{cmH}}_{2}\text{O}$ after the closure cascade to the state described above.
— Full closure occurs almost exclusively at the terminal generation, but with multiple branches (by subtended closure fraction) between the trunk and terminal generations.

Together figures 1 and 2 illustrate that we have captured the essential qualitative and quantitative features selected. There is one remaining quantitative difference, which is that the open branches, post-cascade, sit near $r/r_{0}\approx 0.6$ rather than the targeted 0.4. Given the size of the parameter space, and the fact that this level gives more reasonable resistance ratios (post-constriction/pre-constriction), we elected not to pursue the matter further.

Finally, we observed that the bifurcation onset was sensitive to the tree size. The onset results are shown in figure 3, showing 10 randomized simulations at each tree size (grey) along with the mean (black), as the tree is increased from 6 to 12 generations. This sensitivity is particularly salient owing to the temptation to use smaller trees for parametric calibration, given the computational cost of larger simulations, and also the interpretation of the 12-generation tree as either a sub-tree, or a scaled whole lung (see Discussion).

**Figure 3.**
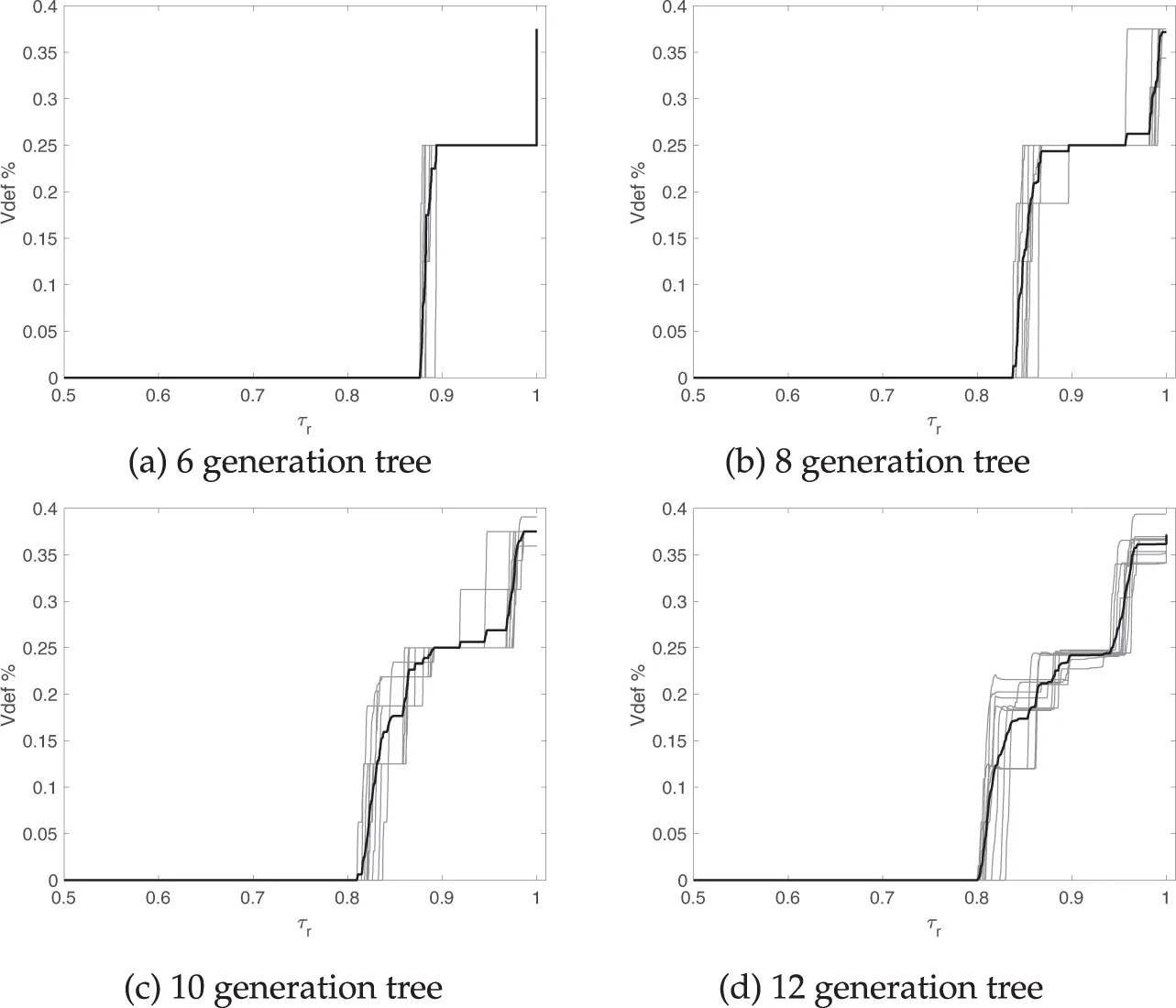
Bifurcation onset variation as tree size is varied. Individual simulations (10) shown in grey, and the mean in black; note that while the overall ventilation defect percentage at $\tau_{r}=1$ does not strongly vary, the bifurcation onset shifts left as the tree depth increases. (a) Six-generation tree. (b) Eight-generation tree. (c) Ten-generation tree. (d) Twelve-generation tree.

## 4. Discussion and conclusion

Here, we have reconstructed and made publicly available for the first time full details and open-source code for the VW05 model lineage. This is based on the central idea of the extension of the Anafi–Wilson terminal airway unit bistability, extended to a bronchial tree under VCV; it is well known and has been cited extensively both in the physiology literature and beyond. Though other models based on the same concept do exist, key details for reproducing the original model have never been published, and code has never been publicly available. Here, we have reconstructed the missing model details and made reproduction code available for the first time, along with demonstrations of reproductions, and several new insights which became apparent during the reproduction process.

There is an inherent tension between the model scale (12 symmetric generations and 4096 terminal units) and the scale of a pair of human lungs; the latter, per Weibel's symmetric model A, would have 65 536 terminal units. Nonetheless, the VW05 model imposes a full 600 ml tidal volume (a typical-to-slightly-large value *in vivo*) on this restricted tree; as a result, the acinar per-unit elastance cannot literally represent a single physical acinus without imposing pressure swings which are far too large. To resolve this, we treat per-unit elastance as a fit parameter, and find $E_{\text{unit}}=77.5$ cmH${}_{2}$O ml^–1^, derived from the $V_{T}=600$ ml anchor and the $\tau_{r}=0.5$ plateau anchor (mean $P_{ao,p}\approx 7\;{\text{cmH}}_{2}\text{O}$ above PEEP) simultaneously. The end result is in excellent qualitative and quantitative agreement with previously published results, but with the caveat that various scales are not strictly compatible: from a tidal volume standpoint, the model is a full lung; from a $V_{\text{TLC}}$ (and elastance) perspective, it is about one-fifth of a lung; geometrically, it is about one-sixteenth of a lung. It is likely that this could be resolved either by scaling up the model to full *in vivo* scale, which was probably computationally prohibitive in 2005 but is tractable now, or by explicitly treating the modelled section as only a portion of the lung. However, the latter introduces additional complications in terms of interpreting PEEP at the trunk, which would no longer be at the mouth but instead mid-lung. We have not attempted either resolution here but confined ourselves to reproduction of the original model and results.

## Data Availability

The MATLAB/Octave and Python implementations described here are openly available at [34].

## Footnotes

^1^
Though the symbol τ is often used for the tethering term, here, we have changed notation to avoid confusion with the muscle activation parameter $\tau_{r}$.

^2^
Unfortunately, there are two different uses of the word ‘bifurcation’ in this paper, inherited separately from physiology and mathematics. In physiology, a bifurcation is of course where the parent airway divides into its daughters; in mathematics, it is where a solution changes qualitatively from one type to another—in this particular instance, where the solution changes from homogeneous constriction to having ‘open’ and ‘closed’ branches. Both uses are too well established to allow alternative terminology, but the distinction should be clear from context.

^3^
The concept of *Thévenin source* arises from treating the airway system as an analogue of an electrical circuit, as is relatively common, and then making use of the attendant engineering machinery; for more details, see e.g. [30].

^4^
See footnote 2.

^5^
c.f. strict ref [2] f=0.16. The original value can be used at the cost of shifting the bifurcation to $\tau_{r}\approx 1.1$ in the 12-generation tree.

^6^
c.f. strict ref [2] $E_{\text{unit}}=255$ cmH${}_{2}$O/mL.

^7^
Although the original implementation states explicit Euler at 10 ms timestep, this is near the stability boundary for the explicit method and the implicit approach is much more robust at minimal extra computational cost.

^8^
A rough estimate suggests that this would have been a 5–10 hour calculation on 2005 hardware, plausible for the original study.
